# 

**Published:** 1868-03-15

**Authors:** 


### CONTENTS:

Hygienic Treatment of Patients. By J. Little, M.D., Le Roy, Ill. (Concluded.) ... 187
Case of Paraplegia. By Chas. M. Clark, M.D ... 195
A Suspension Splint, etc. By E. A. Clarke, M.D., St. Louis ... 198
Senile Gangrene. By Jas. T. Newman, M.D ... 202
Medical Erudition ... 205
Proceedings of the Chicago Medical Society ... 207

BOOK NOTICES:

The Diagnosis, Pathology and Treatment of Diseases of Women ... 212
Proceedings of the American Pharmaceutical Association ... 213
Consumption in New England and elsewhere ... 213
Report Presented to the Ninth Annual Meeting of the New Sydenham Society, held at Dublin, Aug. 10, 1867 ... 214
The Good Man's Legacy; Consumption; Education of the Heart, etc ... 214
Constitution, By-Laws and Code of Ethics of the Chicago College of Pharmacy ... 214

EDITORIAL ... 215

LOOT ... 216

